# Preliminary validation of FastaReada as a measure of reading fluency

**DOI:** 10.3389/fpsyg.2015.01634

**Published:** 2015-10-27

**Authors:** Zena Elhassan, Sheila G. Crewther, Edith L. Bavin, David P. Crewther

**Affiliations:** ^1^Department of Psychology and Counselling, La Trobe UniversityBundoora, VIC, Australia; ^2^Brain Sciences Institute, Swinburne UniversityHawthorn, VIC, Australia

**Keywords:** FastaReada, reading fluency, reading development, assessment, automaticity, visual word recognition, phonological awareness

## Abstract

Fluent reading is characterized by speed and accuracy in the decoding and comprehension of connected text. Although a variety of measures are available for the assessment of reading skills most tests do not evaluate rate of text recognition as reflected in fluent reading. Here we evaluate FastaReada, a customized computer-generated task that was developed to address some of the limitations of currently available measures of reading skills. FastaReada provides a rapid assessment of reading fluency quantified as words read per minute for connected, meaningful text. To test the criterion validity of FastaReada, 124 mainstream school children with typical sensory, mental and motor development were assessed. Performance on FastaReada was correlated with the established Neale Analysis of Reading Ability (NARA) measures of text reading accuracy, rate and comprehension, and common single word measures of pseudoword (non-word) reading, phonetic decoding, phonological awareness (PA) and mode of word decoding (i.e., visual or eidetic versus auditory or phonetic). The results demonstrated strong positive correlations between FastaReada performance and NARA reading rate (*r* = 0.75), accuracy (*r* = 0.83) and comprehension (*r* = 0.63) scores providing evidence for criterion-related validity. Additional evidence for criterion validity was demonstrated through strong positive correlations between FastaReada and both single word eidetic (*r* = 0.81) and phonetic decoding skills (*r* = 0.68). The results also demonstrated FastaReada to be a stronger predictor of eidetic decoding than the NARA rate measure, with FastaReada predicting 14.4% of the variance compared to 2.6% predicted by NARA rate. FastaReada was therefore deemed to be a valid tool for educators, clinicians, and research related assessment of reading accuracy and rate. As expected, analysis with hierarchical regressions also highlighted the closer relationship of fluent reading to rapid visual word recognition than to phonological-based skills. Eidetic decoding was the strongest predictor of FastaReada performance (16.8%) followed by phonetic decoding skill (1.7%). PA did not make a unique contribution after eidetic decoding and phonetic decoding skills were accounted for.

## Introduction

Fluent reading is characterized by speed and accuracy in the decoding of connected text (Fuchs et al., 2001). Although reading fluency is regarded as a key component in the maturation of reading skill (e.g., Samuels, 2006), formal consideration of the construct remains limited (e.g., Kame’enui and Simmons, 2001; Kuhn et al., 2010; Valencia et al., 2010). Furthermore, few studies have attempted to clarify the factors facilitating rate and mode (i.e., visual or eidetic versus auditory or phonetic) of word decoding in fluent reading of comprehensible text. In the current study we assessed FastaReada (Hecht et al., 2004), a computer-based measure of reading fluency by comparing children’s performance on the measure with scores for reading accuracy, comprehension, and rate on a well-established test of text reading ability, the Neale Analysis of Reading Ability (NARA; Neale, 1999) and single word reading on the dyslexia Determination Test (DDT; Griffin and Walton, 1987). To demonstrate criterion-related validity, evidence that FastaReada performance assesses the core features of fluency that is strong, positive correlations with established measures of text reading rate, accuracy and comprehension was sought (Petscher and Kim, 2011). Before discussing the new measure, we discuss our understanding of fluent reading and factors that influence it.

Fluent reading is a multifaceted cognitive process that is usually considered to be dependent on the development of numerous endogenous skills such as phonological awareness (PA; e.g., Ziegler and Goswami, 2005), letter knowledge (e.g., Blaiklock, 2004), visual recognition (e.g., Sereno and Rayner, 2003), attention (e.g., Kinsey et al., 2004), working memory (e.g., Daneman and Carpenter, 1980), naming speed (e.g., Logan, 1997) and speed of processing (e.g., Breznitz and Misra, 2003). Exogenous factors, such as text characteristics, purpose for reading and reading topic have also been shown to contribute to reading fluency (refer to Hosp and Suchey, 2014 for a review).

Following the early work of Vellutino (1977, 1979; e.g., learning to read has most often been associated with and attributed to competence in PA). PA is usually defined as the ability to deconstruct spoken words into distinctive sounds, or phonemes (the distinctive sounds of a language), syllables, and onsets and rimes (e.g., for ‘bat’: /b/onset and [æt] rime) (Liberman, 1971; Treiman and Zukowski, 2013). When alphabetic orthographic systems have a high degree of consistency between grapheme, or letter and phoneme correspondence (Ehri, 1992), phoneme–grapheme knowledge, which is underpinned by PA, provides learner readers with a basic strategy for decoding printed text into its spoken form (Castles and Nation, 2006). However, dependence on this slow and laborious approach to reading, which is termed phonetic decoding, gradually decreases as learner readers acquire orthographic and vocabulary skills that facilitate rapid visual recognition of printed words and subsequent fluency (Ehri and Wilce, 1980, 1985; Thomson et al., 2006; Vellutino et al., 2007). Furthermore, alphabetic systems differ in their orthographic complexity; for example, many English words are irregular words that do not conform to typical grapheme–phoneme mapping rules (e.g., *yacht* and *colonel*). Such words necessitate visual recognition for accurate decoding (Boder, 1973; Castles and Coltheart, 1993). Whilst the relationship between PA and reading skills for list words has been widely studied (e.g., Wagner and Torgesen, 1987; Del Campo et al., 2015), the role of PA in fluent reading is not well researched.

Word recognition occurs when the visual representation of a word corresponds with a stored phonological representation in the mental lexicon (Taft, 1986). With practice, whole word recognition, and hence single word and text reading becomes progressively faster, seemingly automatic and reflex-like (Meyer and Felton, 1999; Hecht et al., 2004; Laycock and Crewther, 2008). Fluency in reading is less demanding of cognitive resources than conscious decoding and therefore frees up attentional stores for higher level processing, that is, comprehension (La Berge and Samuels, 1974; Perfetti et al., 1988). In support, numerous studies have shown strong, positive correlations between reading rate and comprehension (Breznitz, 1987; Jenkins et al., 2003; O’Connor et al., 2010). Thus any new tool for assessing reading must be shown to correlate well with assessments that test accuracy of comprehension.

Most available tools for assessments of reading ability operationalize reading fluency as words correctly identified per minute (WCPM) minus errors such as mispronunciations, substitutions, omissions and insertions (Valencia et al., 2010). The Gray Oral Reading Test (GORT) and the Kaufman Test of Educational Achievement (KTEA) provide specific scores of “reading fluency” via a WCPM score. The KTEA utilizes single word list stimuli rather than connected text, which reduces its criterion validity, as fluent reading is often used to acquire meaningful information from connected text. A further disadvantage of single-word reading rate measures is that they are not effective for the identification of children with specific reading comprehension deficits whereas rate of reading for connected text has been shown to differentiate children with and without comprehension deficits (Cutting et al., 2009). While both the GORT and KTEA are marketed as providing measures of fluency, only the GORT and the NARA utilize connected text stimuli. The NARA does not claim to measure fluency; however, its rate score is representative of words read accurately per minute *out loud*, making it akin to the GORT as a measure of fluency. The GORT, KTEA, and NARA all provide a measure of reading comprehension.

Further disadvantages of the GORT, KTEA, and NARA are related to their availability to educators. Such assessments are restricted to professionals trained in the administration and interpretation of norm-referenced standardized tests. Furthermore, the standardized nature of the reading material used in such assessments is likely to increase repeated administration effects (i.e., practice effects) if used frequently. These factors render such tests as inappropriate for regular monitoring of reading skill development, an important strategy for the development of personalized learning plans (Deno, 2003). Long term established benefits of regular monitoring of reading progress include improved learning outcomes, enhanced educator decision-making and increased student awareness of their own performance (e.g., Fuchs and Deno, 1991; Fuchs and Fuchs, 2002).

In order to enable educators to confidently monitor student reading development progress we have developed a customized computer-generated task called FastaReada. FastaReada has been designed to provide a quick and reliable measure of rate and accuracy of text reading. The defining feature of this task that sets it apart from other WCPM measures is that it utilizes a maximum-likelihood parameter estimation by sequential testing (PEST) testing method to establish the threshold exposure time required to decode short pieces of text (six words). Testing begins with a long exposure time that typically developing students can easily verbalize. With each correct response verbalized, the exposure time becomes shorter, encouraging fluent readers to read silently whilst text is exposed, and then to repeat the words after they disappear. This technique requires encoding of words visualized prior to verbalization, ensuring that FastaReada can also test aspects of working memory, and the cognitive speed of reading by reducing the impact of motor limits on verbal reaction times (Swanson et al., 2009).

The current research compared FastaReada performances with reading accuracy, rate, and comprehension scores on the well-established test of reading ability, the NARA to meet the requirement of criterion-related validity that is, strong, positive correlations between all variables. To address shortcomings in the literature, the current research also aimed to examine the contribution of PA, as measured by the Comprehensive Test of Phonological Processing, (CTOPP) to fluent reading. In addition, the contributions to fluent reading from phonetic decoding skills, as measured by the Pseudoword Decoding subtest of the Wechsler Individual Achievement Test, and relative ability to eidetically recognize words compared need phonetic decoding, as measured by the Dyslexia Determination Test, were also investigated. It was hypothesized that:

(1) FastaReada scores would be strongly associated with NARA measures of reading accuracy, comprehension and rate.
(2) Visual word recognition would be a stronger predictor of FastaReada performance than phonetic decoding skill and PA.
(3) The association between FastaReada scores and visual word recognition would be stronger than the association between NARA rate scores and visual word recognition.

## Materials and Methods

### Participants

This study was approved by the La Trobe University Human Ethics Committee (FSTE HEC 13/R22). Consent to collect data from schools was also provided by the Victorian State Department of Education (2012_001425) and Catholic Education Melbourne (GE12/0009 1765). One hundred and twenty-nine children between the ages of 9–12 years were recruited from 3 year levels (Year 4–6) through four mainstream schools in the North East Metropolitan region of Melbourne, Australia. The schools covered regions of high and low socioeconomic conditions. Participant information and consent forms were dissemination to parents and legal guardians of children in the target year levels. Every child who returned a signed consent form was permitted to undergo the entire battery of tests to avoid leaving children with a sense of exclusion. However, for inclusion in data analysis participants required a score above the 10th percentile on the Raven’s Coloured Progressive Matrices (RCPM: a test of non-verbal reasoning ability; Raven et al., 1998), adequate or adequately corrected vision and hearing, and typical sensory, mental and motor development. Three children were excluded from analysis for scoring at or below the 10th percentile on the RCPM. A further two were excluded on the basis of teacher report of confirmed or suspected neurodevelopmental disorder. The final number of participants was 124 (see **Table 1** for demographics).

**Table 1:** Mean, minimum and maximum age of children (in years and months), and number of each sex in each year level.

	Mean age	Minimum age	Maximum age	Male	Female
Year 4	9;11	9;1	10;5	17	25
Year 5	10;10	10;2	11;8	18	23
Year 6	12;0	11;4	12;10	20	21
**All year levels**	10;11	9;1	12;0	55	69

### Materials

#### FastaReada (Hecht et al., 2004)

FastaReada is a customized computer-generated task designed using VPixx (www.VPixx.com) that measures reading fluency. An excerpt from a contemporary novel, which appeals to children between the ages of 9–12 years of age (permission received from Penguin Group) is presented in narrative order, six words at a time. The presentation time for the group of words presented (Lucida Grande font, 60 pt) was controlled via the PEST adaptive staircase algorithm based on a maximum-likelihood threshold estimation. Children were asked to read the stimulus out loud as accurately as possible. The investigator indicated accurate or inaccurate decoding at the end of each trial. Prior to assessment with FastaReada children were warned that the duration of stimuli presentation would eventually become so short that they would not be able to read all six words out loud. They were encouraged to attempt each trial in spite of the brief exposure time.

#### Neale Analysis of Reading Ability–Third Edition (NARA-3; Neale, 1999)

The NARA-3 is commonly used in school and clinical settings as a standardized measure of reading achievement and diagnostic test. It provides objective measures of reading accuracy, reading comprehension, and reading rate in children aged from 6 to 12 years and over. Administration takes approximately 20 min (Neale, 1999). The NARA-3 was administered according to the standard procedure for testing, as outlined in the NARA-3 manual (Neale, 1999).

In summary, children were instructed to read a series of prose passages presented in book form and answer questions about each passage at its conclusion. Each passage was accompanied by a line drawing that was intended to set the scene rather than to provide detail. The investigator corrected and recorded the number of errors, including mispronunciations, substitutions (i.e., real words used instead of the word in the passage), refusals (i.e., child pauses for approximately 4–6 s and does not make an attempt at the word), additions (i.e., child inserts words or part of words into the passage), omissions (i.e., child omits words from the passage), and reversals (e.g., child says ‘no’ for ‘on’). The investigator also recorded the time the child took to read each passage and marked the child’s answers to questions as correct or incorrect. There were six passages in total, which were presented in order of increasing difficulty. Testing was discontinued after the child reached the ceiling for reading errors in a passage (16 errors for passages 1–5 or 20 errors for passage 6). Separate scores for accuracy, comprehension and reading rate were obtained.

The NARA-3 has been shown to be a reliable and valid tool for the assessment of accuracy, rate and comprehension of oral reading skills. Reliability results for the NARA-3 ranged from moderate to high levels of internal consistency across groups based on years of schooling (0.91–0.96 for accuracy, 0.71–0.96 for comprehension, and 0.73–0.96 for rate (Neale, 1999). The assessment has been shown to have high content and face validity for the construct of oral reading (Neale, 1999). Additionally, it has been shown to have criterion-related validity through its significant correlations with other tests of reading skills (e.g., Moorehouse and Yule, 1974) and through its efficacy at predicting future reading ability (McKay, unpublished as cited in Neale, 1999). Finally, the positive correlation between score and years of schooling provides evidence of construct related validity (Neale, 1999).

#### Dyslexia Determination Test (DDT; Griffin and Walton, 1987)

The DDT is a diagnostic assessment tool that is used to identify the nature, type, and severity of an individual’s learning difficulties (Wesson, 1993). The DDT is designed for students between Year 2 to Year 12 levels and consists of three sections that assess single word reading, writing, and spelling abilities of children (Wagner et al., 1999). The current study utilized the DDT decoding subtest to identify the degree of visual recognition and phonetic decoding strategies used by each child when reading word lists. The DDT was administered according to the standard procedure for testing (Wagner et al., 1999).

Children were asked to commence orally decoding the list words from the initial list (i.e., the pre-primer words) rather than the suggested two to three levels below their year level in order to avoid frustration and to assist with building confidence. The items on the list alternate between phonetically irregular words (i.e., requiring visual recognition for accurate decoding) and phonetically regular words (i.e., conforming to English letter-sound rules), In line with the standard DDT procedure, words read correctly within 2 s were marked as eidetic (i.e., visually recognized) on the DDT record form as the rapid response is indicative of visual recognition. Words read correctly after a delay of more than 2 s but within 10 s were considered to require phonetic decoding indicating the use of phonics, syllabication and/or structural analysis in word decoding. Words that were not read within 10 s, read incorrectly or not attempted were marked as unknown.

#### Pseudoword Decoding: Wechsler Individual Achievement Test–Second Edition (WIAT-II)

The Pseudoword Decoding subtest of the WIAT-II was used to measure phonetic decoding skills. It consists of 54 non-word items, all of which conform to letter-sound rules of regular English words, making the task similar to encountering and decoding unfamiliar words. The investigator administered the task in line with the general assessment procedure (Wechsler, 2007).

In summary, the investigator asked the children to read each item on the Pseudoword Card, from left to right. All children began at the same starting point and the discontinue rule was met once seven consecutive incorrect responses were made.

The WIAT-II is a well-established test of individual achievement. The most reliable and valid measures attained by the WIAT-II are the composite scores; however, the degrees of reliability and validity across individual tests have been shown to be adequate. The Pseudoword Decoding subtest has been shown to be a reliable measure of non-word decoding skill, with high-level inter-item reliability (0.89–0.98) and test–retest stability (0.93) across ages 6–19 years. Evidence of construct- and criterion-related validity has also been demonstrated across the subtests (Wechsler, 2007)

#### Phonological Awareness: Comprehensive Test of Phonological Processing

Participants completed the two PA subtests available for their age group on the CTOPP. The results from these two tasks formed the PA composite score. The subtests assessed elision (the exclusion of one or more sounds from a word) and sound blending (the ability to build whole words by blending individual sounds together). Both PA subtests were administered according to the standard procedure (Wagner et al., 1999).

The PA subtests required the investigator to provide feedback for practice items and the first three test items. Each item could be repeated one additional time if requested by the child. Testing was discontinued following three consecutive incorrect responses. For the elision subtest the investigator asked the child to say a compound-word. After the word was verbalized, the investigator asked the child to say the word again without one of the segments (e.g., “Say *steamboat* without saying *boat*”). For the sound blending subtest children were instructed to listen carefully as words were voiced in small parts, one part at a time, and then to put the parts together to verbalize the whole word (e.g., “*What word do these sounds make when you put them together c-o-m-p-u-t-e-r?*”). Data analysis was conducted with raw composite scores.

The CTOPP has been shown to be a reliable and valid tool for the assessment of PA. Wagner et al. (1999) demonstrated moderate to high internal consistency for all subtests across groups based on age (specifically 0.81 to 0.92 for Elision and 0.78 to 0.89 for Sound Blending). Reliability was also high for time sampling and inter-scorer differences (Wagner et al., 1999). Strong correlations have been demonstrated between the PA composite of the CTOPP and the Lindamood Auditory Conceptualization Test (Wagner et al., 1999), Woodcock Reading Mastery Tests – Revised (Wagner et al., 1994, 1997), as well as the Test of Word Reading Efficiency (Wagner et al., 1999), providing support for criterion-prediction validity. Additionally, construct validity of the CTOPP is demonstrated by the positive correlation between age and score, and the test items are sufficiently correlated for the verification of content validity (Wagner et al., 1999).

#### Raven’s Colored Progressive Matrices (RCPM) Test

The RCPM (Raven et al., 1998) was used to provide a standardized, untimed, non-verbal measure of general intelligence through the assessment of non-verbal reasoning ability. The RCPM has been norm-referenced in numerous countries, including Australia, and earlier work has shown that the RCPM is an appropriate measure for typically developing children aged 5 to 11 years, as well as children with reading and/or learning disorders (Cotton et al., 2005b).

The RCPM consists of three sets of 12 colored multiple-choice items that gradually increase in complexity (A, Ab, B). Each item is presented on an A4 sized sheet of paper and consists of an incomplete matrix. Children were asked to identify or point to one of six figures positioned below the rectangle that would correctly complete the pattern. A score of one point was rewarded for each correct answer, whilst incorrect answers scored a zero. The scores were tallied upon completion of the task to provide an overall raw score. Raw scores were converted to percentile scores to rank non-verbal intelligence on the basis of chronological age.

The RCPM has been demonstrated to have good test-retest reliability at *r* = 0.80 (Raven et al., 1998) and high internal consistency (*r* = 0.89), with minimal variation across age levels (Cotton et al., 2005b).

### Procedure

Testing was conducted over three sessions that ran for approximately 30 min each, in order to reduce disruptions to classroom learning. The testing sessions were run during school hours, in a quiet room within the child’s school. The order of tests was determined in an order that would promote interest and reduce fatigue (i.e., cognitively demanding and paper-based tests were limited in each session and computer-based tests were administered toward the end of each session to act as an incentive).

Prior to the commencement of each session, each child was asked “Would you like to play some paper and computer games with us?” and encouraged to request breaks or tell the investigator if they wanted to stop participating. All children recruited stated that they wanted to participate and there were no requests made for breaks or termination of participation. Children were praised for their performances at the conclusion of each session and were encouraged to choose a “thank you gift” from a box of novelty stationary items.

### Statistical Procedures

Data was screened for accuracy of entry, missing values and violations of the assumptions of statistical tests, prior to statistical analysis using Statistical Package for Social Scientists (IBM SPSS Statistics 22). The data set was deemed to be accurate and free from missing values. Preliminary analyses of all data were conducted to assess the assumptions of homoscedasticity, linearity and homogeneity of variance. The frequency distribution of each variable was assessed for violations of normality using standardized indices (z) of skewness and kurtosis with a conservative criterion of *α* = 0.001; half the variables were considered close to normal, with skewness and kurtosis values falling between -6.56 and +9.47. Outliers identified for the variable FastaReada were rescored to the next lowest score identified to reduce influence on remaining data (Tabachnick and Fidell, 2013). A square root transformation was then applied to the FastaReada variable. Square root transformations resulted in substantial improvement for variables in violation of normality, including FastaReada, PA, eidetic decoding, Pseudoword decoding, and NARA-3 accuracy. Reflected transformations were applied to NARA-3 accuracy, eidetic decoding, pseudoword decoding, and PA variables (Tabachnick and Fidell, 2013). No interactions were found between the variables.

Pearson product moment correlations and hierarchical regressions were used to explore the data. Correlation coefficients (*r*) are reported to quantify the degree and direction of the relationships between variables with 0.10–0.29 considered a weak relationship, 0.30–0.49 a medium relationship, and 0.50–1.0 a strong relationship (Cohen, 1988, pp. 79–81). Hierarchical regression was used to explore the proportion of variance in the dependent variables that could be accounted for by one or more independent variables (i.e., how well the independent variables predicted the dependent variable). Change in multiple correlation coefficient squared (*R*^2^) values were reported on a range from 0 to 100% to indicate the proportion of variance that was accounted for by each set of independent variables. Squared semi-partial correlations (*Sr*^2^) were used to quantify the unique contributions of individual independent variables.

## Results

### Hypothesis One: Relationship between FastaReada Scores and NARA-3

Bivariate correlations were conducted on FastaReada scores and the scores obtained on the three NARA-3 variables. The relationships between FastaReada performance, and performances on the accuracy, rate, and comprehension subtests were investigated using Pearson product-moment correlation coefficient. Results of the analysis are shown in **Table 2**. Higher NARA-3 accuracy and rate scores were strongly associated with higher FastaReada scores across all year levels tested (*r* = 0.79–0.85 for accuracy and *r* = 0.61–0.82 for rate). Higher NARA-3 comprehension scores were strongly associated with higher FastaReada scores for children in Years 5 (*r* = 0.70) and 6 (*r* = 0.64). Higher Year 4 NARA-3 comprehension scores were moderately positively correlated with rapid and accurate performance on FastaReada (*r* = 0.47).

**Table 2:** Correlations between FastaReada Scores with NARA-3 accuracy, comprehension, and rate subtests for each year level.

	NARA-3		
	Accuracy	Comprehension	Rate
Grade 4	0.79^∗∗^	0.47^∗∗^	0.61^∗∗^
Grade 5	0.81^∗∗^	0.70^∗∗^	0.82^∗∗^
Grade 6	0.85^∗∗^	0.64^∗∗^	0.78^∗∗^
**All Year Levels**	0.83^∗∗^	0.63^∗∗^	0.75^∗∗^

*NARA-3, Neale Analysis of Reading Ability – Third Edition. ^∗∗^p < 0.01. N = 124, Grade 4 n = 42, Grade 5 n = 41, Grade 6 n = 41.*

### Hypothesis Two: Relationships between FastaReada and Eidetic and Phonological Decoding

A preliminary correlation matrix was run in order to assess the associations between visual word recognition, FastaReada performance, phonetic decoding, and PA. The relationships between FastaReada performance and performance on the remainder of the variables were investigated using Pearson product-moment correlation coefficient. Results of the analysis are shown in **Table 3**. The results revealed strong positive correlations between FastaReada scores and scores on the visual word recognition component of the DDT across year levels (*r* = 0.77–0.82). Phonetic decoding ability and performance on FastaReada were also strongly positively correlated (*r* = 0.59–0.76). The correlation between FastaReada performance and PA was moderately positively correlated in Year 5 (*r* = 0.48) and 6 children (*r* = 0.44). However, PA was not shown to be associated with FastaReada performance at a statistically significant level in Year 4 children (*r* = 0.20). A weak to moderate negative correlation was documented between the level of phonetic decoding on the DDT and FastaReada performance (*r* = -0.32 to -0.19). When all year levels were combined, a moderate positive association was shown between FastaReada performance and PA (*r* = 0.37).

**Table 3:** Correlations between FastaReada scores and scores on tests of decoding mode, phonetic decoding skill and phonological awareness for each year level.

	DDT		WIAT-II Phonological decoding	CTOPP Phonological awareness
	Eidetic	Phonetic		
Grade 4	0.82^∗∗^	-0.20^∗∗^	0.59^∗∗^	0.20^∗∗^
Grade 5	0.82^∗∗^	-0.32^∗∗^	0.76^∗∗^	0.48^∗∗^
Grade 6	0.77^∗∗^	-0.19^∗∗^	0.71^∗∗^	0.44^∗∗^
**All year Levels**	0.81^∗∗^	-0.25^∗∗^	0.68^∗∗^	0.37^∗∗^

*WIAT-II, Wechsler Individual Achievement Test – Second Edition; CTOPP, comprehensive test of phonological processing; DDT, Dyslexia Determination Test. ^∗∗^p < 0.01. N = 124, Grade 4 n = 42, Grade 5 n = 41, Grade 6 n = 41.*

Hierarchical regression analysis was conducted to ascertain the contributions of PA, phonetic decoding, and eidetic decoding skills to reading fluency. The regression controlled for the contribution of age and non-verbal reasoning ability to the variance in FastaReada scores in the first step. The second step explored the variance in FastaReada scores attributable to PA and phonetic decoding abilities. The eidetic decoding variable was entered in the final step in order to control for the contributions of phonological-based skills. The results are presented in **Table 4**.

**Table 4:** Hierarchical regression results of non-verbal reasoning, age in years, visual word recognition, phonetic decoding skill, and phonological awareness predicting overall FastaReada performance.

		Final summary			Step summary	
Predictor		*Beta*	*r*	*Sr*	*ΔR^2^*	*p*
Step 1					0.180	*<*0.001
	Age (years)	0.26	0.31	0.26		
	Non-verbal reasoning	0.30	0.34	0.30		
Step 2					0.334	*<*0.001
	Age (years)	0.16	0.31	0.15		
	Non-verbal reasoning	0.15	0.34	0.15		
	Phonological awareness	0.03	0.37	0.02		
	Phonetic decoding skills	0.59	0.68	0.48		
Step 3					0.166	<0.001
	Age (years)	0.03	0.31	0.03		
	Non-verbal reasoning	0.07	0.34	0.07		
	Phonological awareness	0.00	0.37	0.00		
	Phonetic decoding	0.20	0.68	0.13		
	Eidetic decoding	0.63	0.81	0.41		

*N = 124.*

The results revealed that age and non-verbal reasoning (Step 1) accounted for 18% of the variance in FastaReada performance, *F*(2,121) = 13.32, *p* < 0.001. The addition of the phonological-based skills in Step 2 contributed significantly to the regression model, explaining a further 33.4% of the variance in FastaReada performance, *F*(3,119) = 40.99, *p* < 0.001. Finally, the introduction of eidetic decoding skills explained a further 16.6% of the variance in FastaReada performance. The visual word recognition strategy of decoding was the strongest unique contributor to FastaReada performance (16.8%). Phonetic decoding skill also contributed unique variance of 1.7%. Age, non-verbal reasoning and PA did not make unique contributions in the final model. Together, the five independent variables accounted for 68.1% of the variance in FastaReada performance.

### Hypothesis Three: Relationship between FastaReada and Visual Word Recognition and NARA-3 Reading Rate

Bivariate correlations were conducted on visual word recognition scores and the scores obtained on FastaReada and the NARA-3 rate subtest. The relationships between visual word recognition strategy utilization and scores on FastaReada and the NARA-3 rate subtest were investigated using Pearson product-moment correlation coefficient. The results revealed strong positive correlations between both FastaReada and NARA-3 rate scores and visual word recognition strategy utilization. Visual word recognition was more closely associated with FastaReada performance, *r*(122) = 0.81, *p* < 0.001, than with NARA-3 rate performance, *r*(122) = 0.73, *p* < 0.001.

Further analysis using hierarchical regression controlled for the contribution of age and non-verbal reasoning ability to the variance in eidetic decoding in the first step. The second step explored the variance in eidetic decoding attributable to NARA-3 rate and FastaReada scores. The results are presented in **Table 5**.

**Table 5:** Hierarchical regression results of non-verbal reasoning, age in years, reading rate scores and FastaReada scores predicting eidetic decoding.

		Final summary			Step summary	
Predictor		*Beta*	*r*	*Sr*	*ΔR^2^*	*p*
Step 1					0.201	<0.001
	Age (years)	0.31	0.35	0.30		
	Non-verbal reasoning	0.28	0.33	0.28		
Step 2					0.496	<0.001
	Age (years)	0.09	0.35	0.09		
	Non-verbal reasoning	0.03	0.33	0.03		
	Reading rate	0.24	0.73	0.16		
	FastaReada	0.59	0.81	0.38		

*N = 124.*

Age and non-verbal reasoning contributed significantly to the regression model, *F*(2,121) = 15.21, *p* < 0.001, and accounted for 20.1% of the variation in eidetic decoding. The addition of NARA-3 reading rate and FastaReada variables explained an additional 49.6% of variation in eidetic decoding and this change in *R*^2^ was significant, *F*(2,119) = 97.37, *p* < 0.001. FastaReada was the strongest unique predictor of visual word recognition strategy utilization (14.4%). Performance on the NARA-3 rate subtest uniquely predicted 2.6% of the variance in visual word recognition strategy utilization. Together, the five independent variables accounted for 69.7% of the variance in eidetic decoding.

## Discussion

The purpose of this study was to evaluate if FastaReada can be utilized as a valid measure of reading skills. The relationship between FastaReada performance and comprehension was also examined, as was the importance of visual recognition in comparison to phonological skills for fast and accurate reading. The results are discussed in relation to the three hypotheses.

The first hypothesis that FastaReada scores would be associated with scores from the Accuracy, Comprehension and Rate subtests from the NARA-3 was strongly supported. The rate of words read accurately per minute on FastaReada was well correlated with individual subtest results on the NARA-3. Children who scored higher on FastaReada had greater accuracy rates for the words in the assigned prose passages of the NARA-3 reader, greater degrees of understanding of each passage, and were able to decode more words accurately per minute than those who scored lower on FastaReada. The strong and significant correlations obtained in this study between FastaReada and the three subtests on the well-established, well-validated, and reliable measure of reading skills, the NARA-3 (Moorehouse and Yule, 1974; Neale, 1999) indicate that FastaReada meets the test of criterion-related validity. FastaReada thus provides a valid and direct measure of accuracy and speed, the two major accepted components of reading fluency. The findings are also consistent with research proposing that reading speed is a key factor in reading comprehension (Perfetti and Hogaboam, 1975; Jenkins et al., 2003; Danne et al., 2005; Rasinski et al., 2005; Yovanoff et al., 2005). The current results support the notion that conscious attentional demands for decoding inhibit understanding (Shankweiler, 1999; Klauda and Guthrie, 2008; Cutting et al., 2009).

In accordance with the second hypothesis, the contribution of visual word recognition was significantly greater than that of phonetic decoding skills (16.7% compared to 1.7%). These results demonstrate that fluent readers have already acquired the skills to decode novel words with speed and accuracy, and that these skills are predominantly reliant on competence in visual word recognition. Certainly, it has been demonstrated in the current study that the increased utilization of a phonetic decoding strategy, when reading, is detrimental to reading rate. In line with these results, training in phonemic awareness has been shown to improve reading difficulties at the word-level, contributing to more accurate word identification (Torgesen et al., 2001; Hatcher et al., 2006; Bowyer-Crane et al., 2008). However, improvements in single word reading accuracy do not imply improvements in continuous text reading rate. Indeed, slow reading rate often remains into adulthood despite remediation of decoding skills (Torgesen et al., 2001; O’Connor et al., 2007; Laycock and Crewther, 2008). Torgesen et al. (2001) reasoned that a limited repertoire for visually recognizable words results in increased reliance on phonemic analysis or guessing from context for word identification, and that this is inversely related to reading rate.

Findings from the second hypothesis also showed that fast and accurate readers tend to have higher levels of PA and phonetic decoding skills. The results showed a small but significant contribution of phonetic decoding skill to fast and accurate reading (1.7%). However, the results did not support a unique role for PA in fast and accurate reading after controlling for the contributions of visual word recognition and phonetic decoding. PA has been shown to be an important predictor for future reading in preliterate children (Gallagher et al., 2000; Snowling et al., 2003; Puolakanaho et al., 2004). Yet, the predictive nature of PA for reading ability has been documented to decrease with maturation of reading skills. Wagner et al. (1997) examined the changing relationship between PA and reading ability in a large longitudinal study. They documented a decrease in the unique contribution of PA to reading from 23% to only 4% between kindergarten and the fourth year of schooling after the contributions of word reading skills and vocabulary were taken into account. This is likely to reflect a shift toward an increasing reliance on a visual recognition strategy for the decoding of words that have become increasingly familiar with years of practice and the contribution of a growing vocabulary. Thomson et al. (2006) demonstrated similar findings in young learner readers. The findings from the current study reinforce the findings of Thomson et al. (2006).

The final hypothesis that visual word recognition would be more strongly associated with performance on FastaReada than on the NARA-3 was supported. FastaReada was found to be better able to tap into the reader’s efficacy in visual word recognition than the NARA-3 Rate subtest. An important feature of FastaReada is its utilization of an adaptive staircase routine for stimulus presentation. The adaptive staircase algorithm allows FastaReada to determine the shortest exposure time necessary for accurate visual word recognition whilst ensuring reliability of the measure. Stimulus exposure time in FastaReada, which becomes shorter with each correct response, can become so brief with increasing reading skill levels, that the accurate verbalization of text at the time of presentation becomes unachievable due to the motor limits of verbalization. Readers are therefore forced to read silently in order to encode the text and then verbalize them after they disappear. This method allows FastaReada to tap into visual word recognition and memory for series of words as facilitated by discourse-based anticipation. Thus, FastaReada can provide a more accurate measure of reading speed than traditional reading measures as it is not constrained by motor limits associated with the verbalization of text. This feature is particularly pertinent as adult reading is usually performed silently (Miller and Smith, 1989; Kragler, 1995).

The current study has shown FastaReada to be a valid measure of reading fluency through its strong positive correlations with established NARA tests of reading speed and accuracy for connected comprehensible text. Additional support for its criterion validity as a reading fluency measure was obtained through its strong relationship with established measures of eidetic decoding ability and phonetic decoding skills, as well as its moderate to strong relationship with the NARA reading comprehension measure. FastaReada therefore has the potential to play a valuable role in the education system. As an assessment tool that does not require specialist training, FastaReada can be used by educators to screen baseline-reading abilities and to monitor progress in the development of reading skills. By providing an indication of student progress, FastaReada would allow educators to develop and provide more effective, individualized reading programs that promote and nurture the development of reading fluency. FastaReada results can also alert educators and clinicians to the need for further assessment for better identification of the individual’s specific difficulty.

We acknowledge that the current study has associated limitations. Previous studies, including our own (Rutkowski et al., 2003; Cotton et al., 2005a; Alloway, 2006; Laycock et al., 2006; Thomson et al., 2006; Vidyasagar and Pammer, 2010) indicate the important role attention and working memory contribute to reading abilities as regulatory and mediating factors respectively. Thus once validation studies for FastaReada are complete further investigations attention and working memory as contributory variables to FastaReada performance will be beneficial. This future research will be important as FastaReada requires contributions of working memory when stimulus presentation is so short that examinees cannot verbalize the text during the exposure time. Additionally, the current research would have benefitted from a longitudinal design for the examination of the predictive validity of FastaReada. A longitudinal study would have enhanced findings related to the contribution of eidetic and phonological based abilities to reading skills over the course of reading development. In addition to phonological skills, rapid automatized naming tasks have been shown to be one of the best predictors of reading fluency. Future validity studies for FastaReada would therefore benefit from the inclusion of rapid automatized naming tasks in their design. Clearly, the next step required in the development of FastaReada as a measure of reading fluency is the provision of normative data, the determination of appropriate cut-off points for each year level, and possibly the design of a range of appropriate prose passages for the different year levels and for test and retest conditions.

## Conclusion

The current study has tested the criterion-related validity of FastaReada, a brief, computer-generated test of reading fluency that does not require specialist training for administration. FastaReada demonstrated a valid measure of the core features of reading fluency, speed and accuracy, as demonstrated by strong correlations with the established measures of accuracy, rate and comprehension on the NARA. FastaReada performance was also strongly correlated with measures of eidetic and phonetic text decoding abilities that have often been associated with the development of reading skills. FastaReada therefore provides a means for educators, clinicians, and researchers to quickly obtain a measure of reading fluency with relative confidence. The use of such a tool could contribute to more effective individualized reading instruction and remediation through the monitoring of reading fluency development. The current study also drew attention to the rapid, automatic, and visual nature of fluent reading. Results showed that while reading rate and accuracy is associated with PA, PA is not a predictor of fluency when the contributions of visual word recognition and phonetic decoding skills are taken into account. The multifaceted nature of reading skills is becoming increasingly recognized within the literature. This study adds to the burgeoning literature on reading fluency that is branching away from the traditional focus on phonological skills and exploring deficits in areas such as attention, working memory, and visual recognition. Further evaluation of FastaReada is warranted to assess reliability and to determine the most appropriate cut-off points for children of different year levels.

## Conflict of Interest Statement

The authors declare that the research was conducted in the absence of any commercial or financial relationships that could be construed as a potential conflict of interest.
